# Homoharringtonine suppresses tumor proliferation and migration by regulating EphB4-mediated β-catenin loss in hepatocellular carcinoma

**DOI:** 10.1038/s41419-020-02902-2

**Published:** 2020-08-14

**Authors:** Man Zhu, Zhengyan Gong, Qing Wu, Qi Su, Tianfeng Yang, Runze Yu, Rui Xu, Yanmin Zhang

**Affiliations:** School of Pharmacy, Health Science Center, Xi’an Jiaotong University, No. 76, Yanta Weststreet, #54, 710061 Xi’an, Shaanxi P.R. China

**Keywords:** Receptor pharmacology, Drug development

## Abstract

Overexpressed EphB4 conduce to tumor development and is regarded as a potential anticancer target. Homoharringtonine (HHT) has been approved for hematologic malignancies treatment, but its effect on hepatocellular carcinoma (HCC) has not been studied. This study elucidated HHT could restrain the proliferation and migration of HCC via an EphB4/β-catenin-dependent manner. We found that the antiproliferative activity of HHT in HCC cells and tumor xenograft was closely related to EphB4 expression. In HepG2, Hep3B and SMMC-7721 cells, EphB4 overexpression or EphrinB2 Fc stimulation augmented HHT-induced inhibitory effect on cell growth and migration ability, and such effect was abrogated when EphB4 was knocked down. The similar growth inhibitory effect of HHT was observed in SMMC-7721 and EphB4^+^/SMMC-7721 cells xenograft in vivo. Preliminary mechanistic investigation indicated that HHT directly bound to EphB4 and suppressed its expression. Data obtained from HCC patients revealed increased β-catenin expression and a positive correlation between EphB4 expression and β-catenin levels. HHT-induced EphB4 suppression promoted the phosphorylation and loss of β-catenin, which triggered regulation of β-catenin downstream signaling related to migration, resulting in the reversion of EMT in TGF-β-induced HepG2 cells. Collectively, this study provided a groundwork for HHT as an effective antitumor agent for HCC in an EphB4/β-catenin-dependent manner.

## Introduction

Globally, hepatocellular carcinoma (HCC) is one of the most fatal malignancies with poor prognosis and an increasing incidence^1^. Although the major therapeutic approaches such as surgical resection, radiation therapy, and chemotherapy have advanced clinical applications, the 5-year survival rate of HCC remains less than 30%^2^. Most patients still suffer from tumor recur, invasiveness, and metastasis. At present, sorafenib, a multiple tyrosine kinase inhibitor, is one of the most representative options for advanced HCC, but is sometimes limited and accompanied with reduced sensitivity and severe adverse events^3,4^. Therefore, much effort is needed on this front to uncover new anti-HCC therapeutic strategies^5^.

Erythropoietin-producing hepatocyte receptor B4 (EphB4) is a member of the tyrosine kinase family and plays a pivotal role in tumor progression^6–8^. Activated by its corresponding ligand EphrinB2, EphB4 controls cell–cell interactions, angiogenesis, tumor growth, and metastasis^9,10^. Studies on the expression of EphB4 in numerous cancer types have shown overexpressed level in breast, colorectal, lung, and blood cancers correlating with poor prognosis^11–13^. It has been reported that high EphB4 expression enhanced the growth and migration of pancreatic, colorectal and papillary thyroid carcinoma, and such effect could be reversed by EphB4 knockdown, making EphB4 a promising target for cancer treatment^14–16^. Our previous study has confirmed the high expression of EphB4 in HCC^17^, and its function in HCC migration remains poorly understood.

**Fig. 1:**
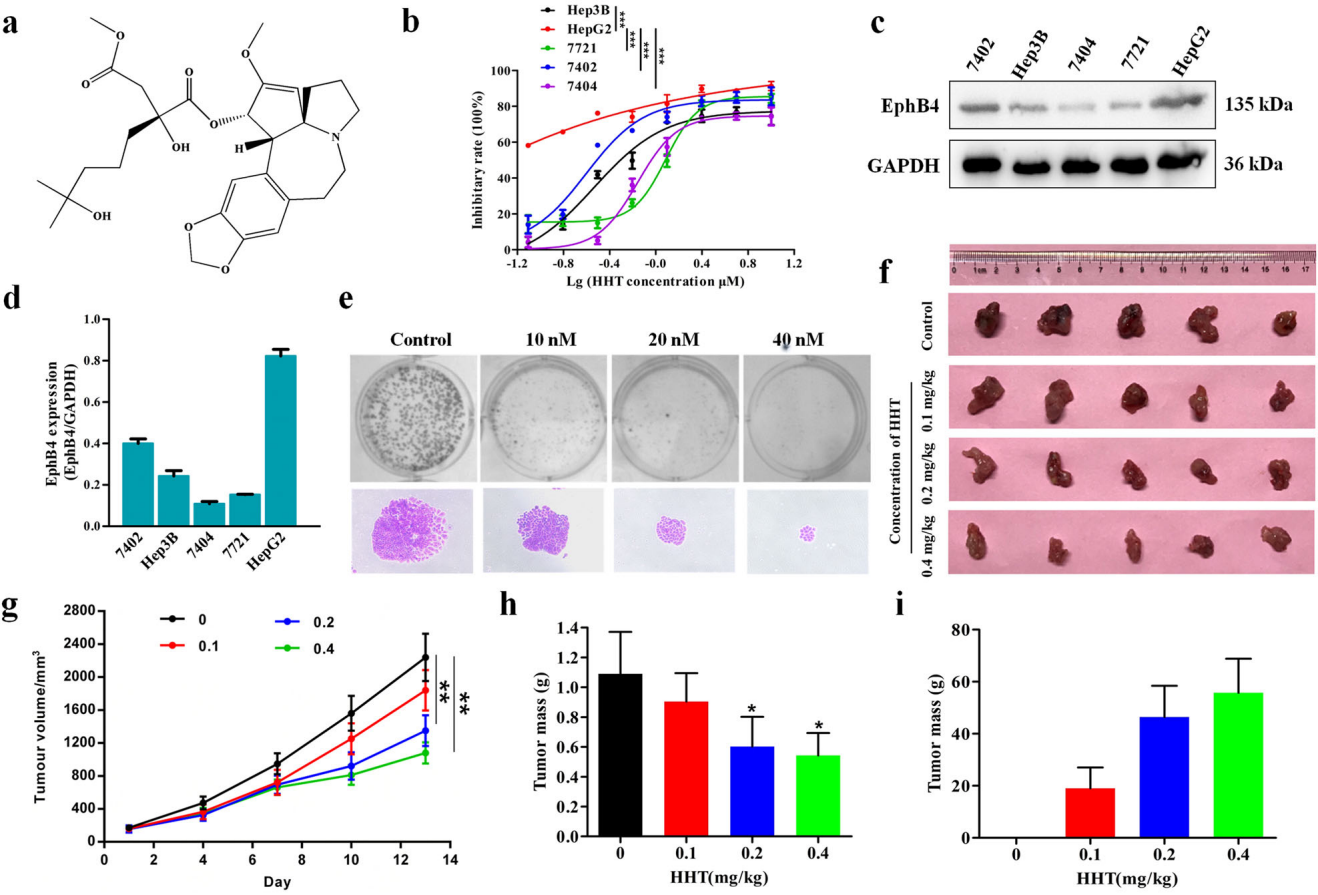
HHT exhibited a growth inhibitory effect on HCC cells in vitro and in vivo. **a** The chemical structure of HHT. **b** Effects of HHT on cell proliferation in Hep3B, HepG2, SMMC-7721 (7721), Bel-7402 (7402), and Bel-7404 (7404) cells were determined by MTT assay. ***p < 0.001 compared to the IC_50_ of HepG2 cells. Cells were treated with increased gradients of HHT for 48 h (*n* = 5 cultures for each dose). **c** Protein expression of EphB4 in Hep3B, HepG2, 7721, 7402, and 7404 cells. **d** Quantification of **c** (*n* = 3 independent experiments). **e** Effects of HHT on colony formation in HepG2 cells. The upper row: the colony formation picture; the lower row: the individual colony picture (×200 magnification). **f** Photographs of control and HHT-treated group tumors (*n* = 5 mice). **g** Tumor volume change throughout the study (*n* = 5 mice). **h** Effect of HHT on tumor inhibitory rate (*n* = 5 mice). **g**, **h** data represent mean ± SEM. *p < 0.05, **p < 0.01; compared to vehicle controls. **i** Inhibitory rate of HHT on tumor mass (*n* = 5 mice).

Homoharringtonine (HHT) (Fig. 1a) is a compound extracted from traditional Chinese medicine and has been approved for the treatment of leukemia by Food and Drug Administration^18^. Previous studies indicated that HHT could suppress protein synthesis essential for cancer survival and induce apoptosis by upregulating the proapoptotic protein Bax and inducing caspase-3-mediated cleavage of PARP^19^. In addition to hematologic tumors, HHT also demonstrated its effectiveness in renal cell carcinoma, colon rectal cancer, and nonsmall cell lung cancer^20–22^. However, the effect of HHT on HCC and the underlying EphB4-related mechanism of action have not been studied. In this study, HHT was found to suppress the proliferation and migration of HCC cells through an EphB4/β-catenin dependent manner.

## Results

### HHT exhibited a growth inhibitory effect on HCC cells in vitro and in vivo

To determine the effect of HHT on the cell viability of HCC cells, several different HCC cells HepG2, Bel-7402, Hep3B, and SMMC-7721 were treated with an increased gradient of HHT for 48 h. The results showed that HepG2 cells were most sensitive to HHT treatment with an IC_50_ value of 0.025 μM, while the IC_50_ values of Bel-7402, Hep3B, Bel-7404, and SMMC-7721 cells were 0.251, 0.291, 0.694, and 1.220 μM, respectively (Fig. 1b). Immunoblotting analysis showed that HepG2 cells exhibited higher EphB4 expression (Fig. 1c, d), suggesting the positive correlation between the inhibitory effect of HHT and EphB4 expression. Similar results were obtained from the colony formation assay. HHT significantly reduced the colony size and the number of HepG2 cells at a dose-dependent manner in comparison to the control group (Figs. 1e and S1a). Moreover, xenografts model of HepG2 cells confirmed the antitumor effect of HHT in vivo. HHT gavage groups showed remarkable reduction in tumor growth (Fig. 1f–i), and the inhibitory rate reached 20, 50, and 55% at the 0.1 mg/kg, 0.2 mg/kg, and 0.4 mg/kg in HHT gavage groups, respectively.

### The inhibitory effect of HHT on HCC cells was associated with EphB4 expression

**Fig. 2:**
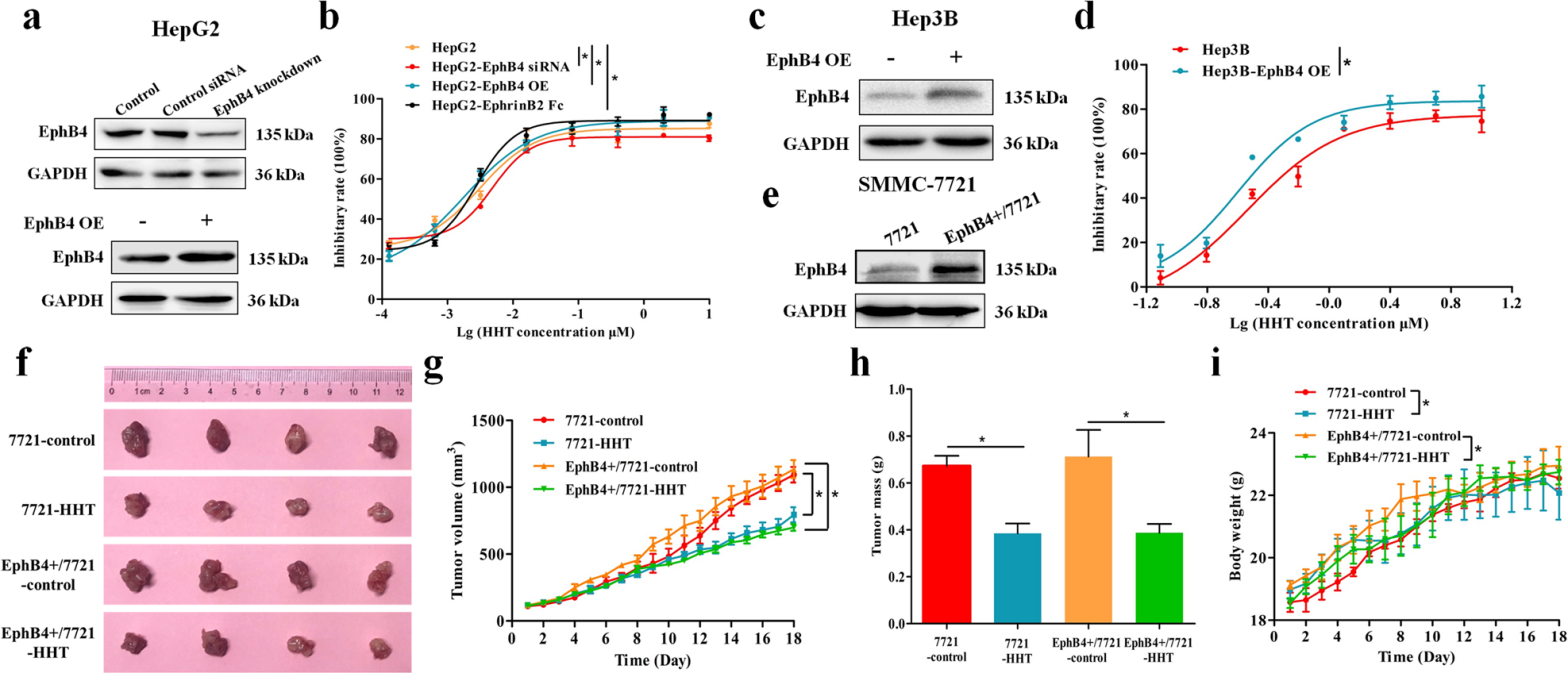
The inhibitory effect of HHT on HCC cells was associated with EphB4 expression. **a** EphB4 expression analysis of EphB4-siRNA or EphB4-overexpression (OE) HepG2 cells. **b** Effects of HHT on cell proliferation in wild-type, EphB4-siRNA, EphB4-OE, or EphrinB2 Fc stimulated HepG2 cells (*n* = 5 cultures for each dose). *p < 0.05 compared to the IC_50_ of HepG2 cells. **c** EphB4 expression analysis of EphB4-OE Hep3B cells. **d** Effects of HHT on cell proliferation in wild-type and EphB4-OE Hep3B cells (*n* = 5 cultures for each dose). *p < 0.05 compared to the IC_50_ of Hep3B cells. **e** EphB4 expression analysis of wild-type 7721 and EphB4^+^/7721 cells. **f** Photographs of control and HHT-treated group of 7721 tumors and EphB4^+^/7721 tumors (*n* = 4 mice). **g** Tumor volume change throughout the study (*n* = 4 mice). **h** Effect of HHT on tumor mass (*n* = 4 mice). **i** Body weight of control and HHT-treated group mice (*n* = 4). **g**–**i** data represent mean ± SEM. *p < 0.05 compared to vehicle controls.

To evaluate whether the proliferation inhibitory effect of HHT on HCC cells was related to EphB4 expression, EphB4 siRNA or plasmid was utilized to transfect the HCC cells (Figs. 2a and S1b), and EphrinB2 Fc was used to stimulate the HCC cells. As is shown in Fig. 2a, b, HepG2 cells with EphB4 knockdown were less sensitive to HHT, whereas HepG2 cells with EphB4 overexpression (EphB4 OE) demonstrated elevated sensitivity to HHT treatment compared with wild type HepG2 cells. HepG2 cells following EphrinB2 Fc stimulation showed a drug response curve that was similar to that of EphB4 OE subline (Fig. 2b). Meanwhile, following transfection with EphB4 plasmid, Hep3B cells harboring high expression of EphB4 showed less cell viability after HHT treatment compared with wild type Hep3B cells (Fig. 2c, d). For in vivo test, an EphB4-overexpressing SMMC-7721 (EphB4^+^/7721) cell line was established (Figs. 2e and S1c) and the anti-tumor effect of HHT on xenograft model of wild type SMMC-7721 (7721) cells and EphB4^+^/7721 cells was investigated. HHT has an enhanced inhibitory effect on EphB4^+^/7721 tumor growth compared with that on wild type 7721 tumor (Fig. 2f–h). And there was no obvious body weight and spleen index reduction during the test (Figs. 2i and S1d).

### The suppression of HHT on SMMC-7721 cells migration was associated with EphB4

**Fig. 3:**
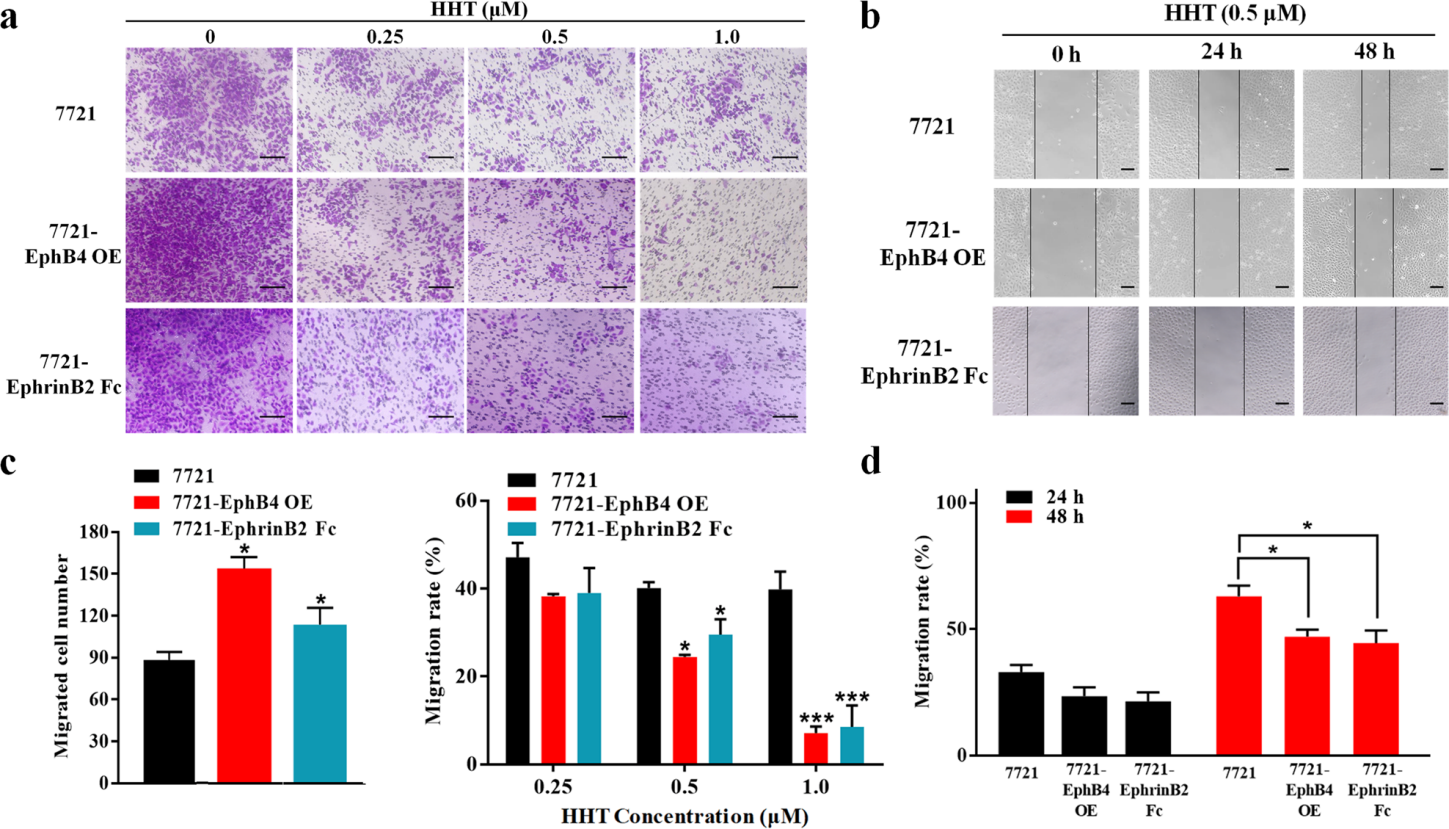
The suppression of HHT on SMMC-7721 cell migration was associated with EphB4. **a** Transwell assays were conducted to observe the migratory cells in HHT-treated wide type 7721, EphB4-OE o,r EphrinB2 Fc stimulated 7721 cells. Scale bars, 100 μm. **b** The migration rate of HHT-treated, EphB4-OE, or EphrinB2 Fc stimulated 7721 cells observed through wound-healing assays. Scale bars, 50 μm. **c** Quantification of **a** (*n* = 5). Left: *p < 0.05 compared to the migrated cell number of 7721 cells; Right: *p < 0.05 and ***p < 0.001 compared to the migration rate of 7721 cells at indicated concentration of HHT. **d** Quantification of **b** (*n* = 5). *p < 0.05 compared to HHT-treated 7721 cells. All data represent mean ± SEM.

Migration assay and wound healing assay were conducted to investigate the effect of HHT on HCC cell migration. The results showed that HHT-treated wide type SMMC-7721 cells had decreased migration as compared with controls, whereas both of EphB4 overexpression and EphrinB2 Fc stimulation in SMMC-7721 cells strikingly enhanced migration restraint effect of HHT (Fig. 3a, c). Similar result was observed in wound healing assay, which demonstrated that both transfection with EphB4 and exogenous stimulation with soluble EphrinB2 Fc in SMMC-7721 cells delayed the closure of wound gaps following HHT treatment (Fig. 3b, d). These results indicated that the suppression of HHT on HCC cells migration was closely associated with EphB4 expression.

### HHT suppressed HepG2 cell migration induced by TGF-β stimulation

**Fig. 4:**
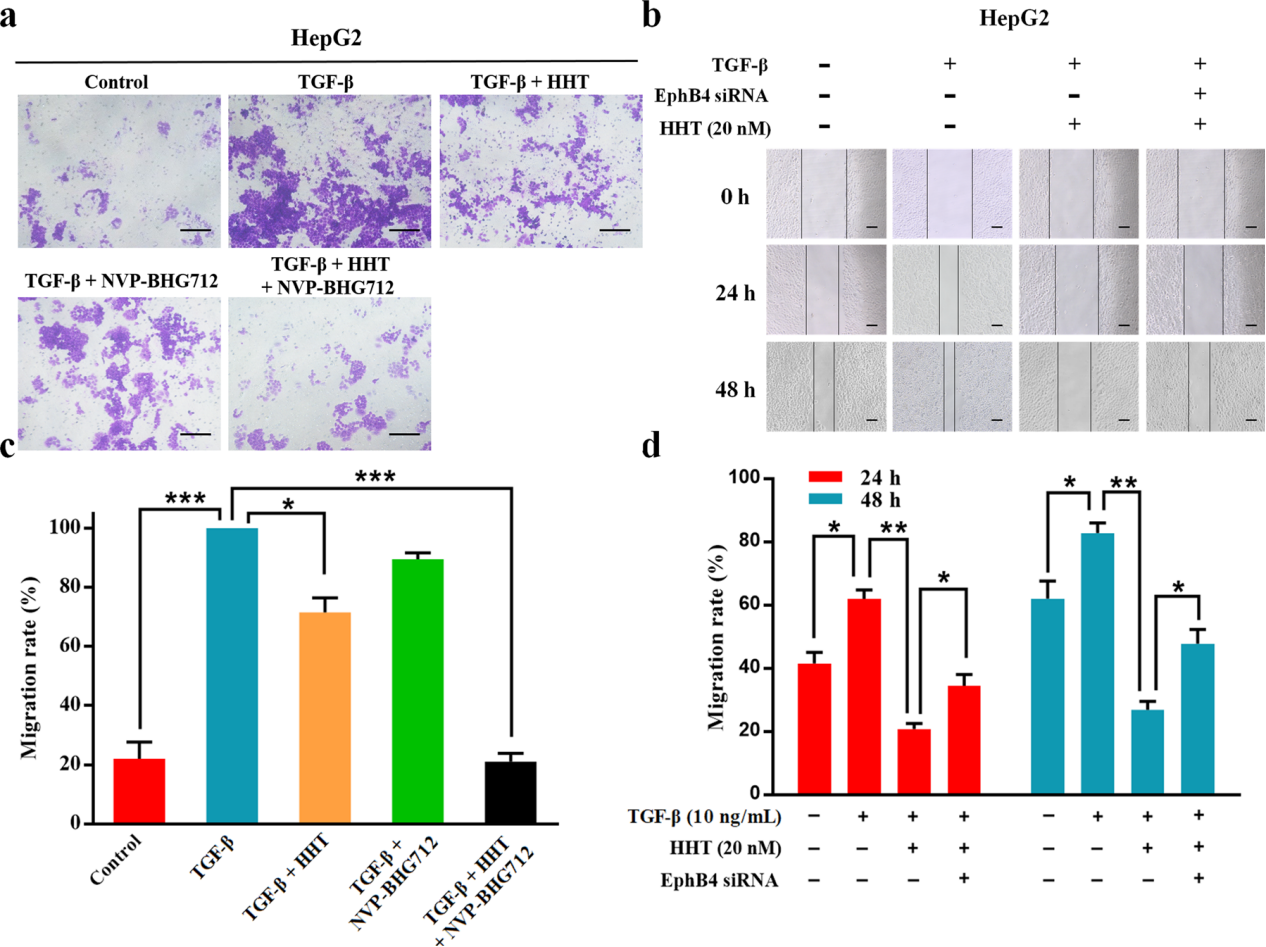
HHT suppressed HepG2 cell migration induced by TGF-β stimulation. **a** Transwell assays were conducted to observe the migratory cells in control and TGF-β, TGF-β + HHT, TGF-β + NVP-BHG712, or TGF-β + HHT + NVP-BHG712 treated HepG2 cells. Scale bars, 100 μm. **b** The migration rate of control and TGF-β, TGF-β + HHT, or TGF-β + HHT + EphB4 siRNA treated HepG2 cells observed through wound-healing assays. Scale bars, 50 μm. **c** Quantification of **a** (*n* = 5). **d** Quantification of **b** (*n* = 5). All data represent mean ± SEM. *p < 0.05, **p < 0.01, and ***p < 0.001; compared to the indicated groups.

TGF-β stimulation could induce EMT and increase the migration of tumor cells. We next investigated the effect of HHT on HCC cells migration after TGF-β stimulation by transwell migration assay and wound healing assay. As shown in Fig. 4a, c, although the higher number of migration cells was observed in the TGF-β induced HepG2 cells, as compared to controls, the addition of HHT reduced the migrated cells. Importantly, concurrent treatment with HHT and NVP-BHG712 (a small molecule EphB4 kinase-specific inhibitor) had a greater restraint effect on the migration of TGF-β induced HepG2 cells. Wound healing assay showed similar results that HHT could delay the closure of wound gaps in TGF-β induced HepG2 cells, whereas the addition of EphB4 siRNA impaired such effect (Fig. 4b, d). These results indicated that TGF-β induced the migration ability in HepG2 cells, which could be abrogated by EphB4 suppression of HHT.

### HHT bound to EphB4 and suppressed its expression

**Fig. 5:**
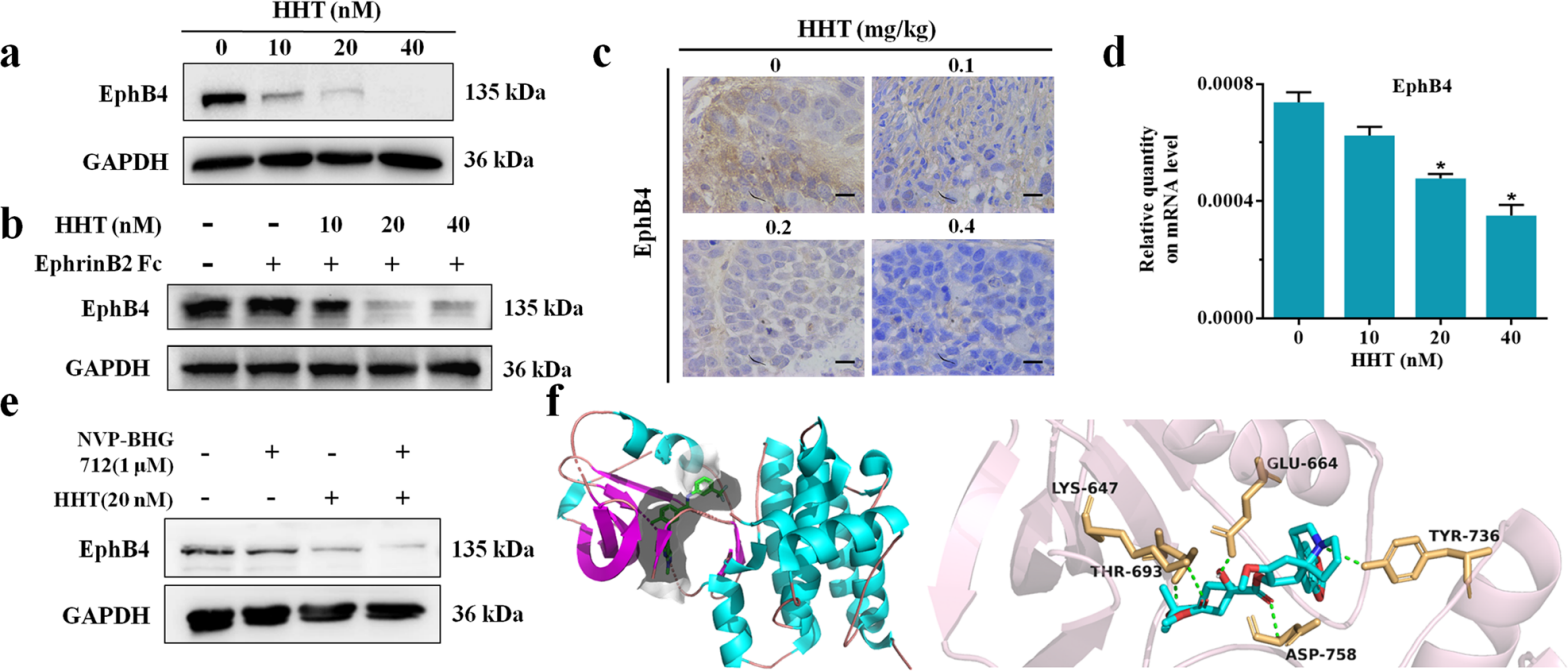
HHT bound to EphB4 and suppressed its expression. **a** Western blot analysis of HepG2 cells EphB4 expression after HHT treatment. **b** Western blot analysis of EphB4 expression in HepG2 cells treated with HHT or/and EphrinB2 Fc. **c** Immunochemistry analysis of EphB4 expression in HepG2 tumors after HHT treatment (*n* = 5). ×400 magnification. **d** RT-PCR analysis of HepG2 cells EphB4 expression after HHT treatment (*n* = 5). All data represent mean ± SEM. *p < 0.05 compared to vehicle controls. **e** Western blot analysis of HepG2 cells EphB4 expression after HHT, NVP-BHG712, or both treatments. **f** Molecular docking analysis of the EphB4 protein and HHT.

We further evaluated the regulation of HHT on EphB4 expression. The results showed decreased EphB4 protein expression after HHT treatment both in HepG2 cells and tumor tissues (Figs. 5a, c and S2a, b). Exogenous stimulation with soluble EphrinB2 Fc increased EphB4 protein expression, while in HepG2 cells treated with EphrinB2 Fc and HHT, the protein levels of EphB4 were strikingly decreased (Figs. 5b and S2c). HHT treatment resulted in a remarkably reduced *EphB4* mRNA level at a dose-dependent manner (Figs. 5d and S2d). We treated HepG2 cells with NVP-BHG712, HHT, or both to evaluate the change of EphB4 expression. The results indicated that co-administration of HHT and NVP-BHG712 produced an even greater decrease in the expression level of EphB4 in HepG2 cells than by either alone (Figs. 5e and S2e). Given these findings, a molecular docking assay was conducted to confirm the affinity of HHT bound to the active site of EphB4. The results revealed that HHT occupied in the active site of EphB4 through five hydrogen bonds which were associated with amino acid residues LYS-647, GLU-664, TYR736, ASP-758, and THR-693 (Fig. 5f). The molecular docking results indicated that HHT fit well with EphB4.

### EphB4 was positively correlated with β-catenin in HCC patients and HHT inhibited the phosphorylation and nuclear translocation of β-catenin

**Fig. 6:**
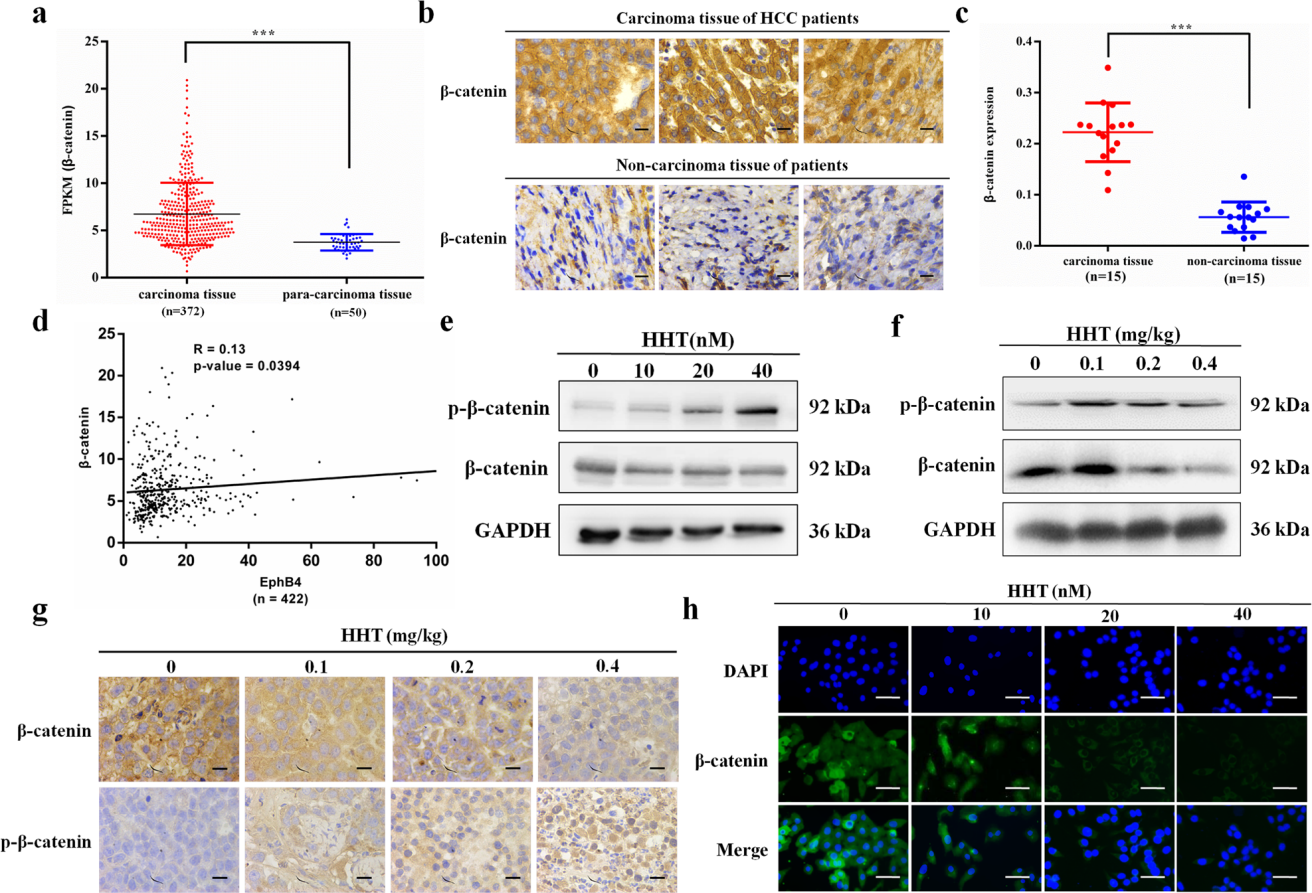
EphB4 was positively correlated with β-catenin in HCC patients and HHT inhibited the phosphorylation and nuclear translocation of β-catenin. **a** mRNA expression of EphB4 in HCC carcinoma tissue and para-carcinoma tissue in the TCGA database (***p < 0.001). **b** Representative figures of immunohistochemical analysis of β-catenin expression in carcinoma and noncarcinoma tissues derived from 15 HCC patients and 15 nonHCC patients, respectively. ×400 magnification. **c** Quantification of **b** (*n* = 15, ***p < 0.001). **d** The positive correlation between the expression of β-catenin and EphB4. **e** Protein expression of β-catenin and p-β-catenin in HepG2 cells treated with HHT for 48 h. **f** Protein expression o**f** β-catenin and p-β-catenin in HepG2 tumor EphB4 expression after HHT treatment. **g** Immunochemistry assay of β-catenin and p-β-catenin in HepG2 tumor tissues. ×400 magnification. **h** Immunofluorescence analysis of the β-catenin protein in HepG2 cells treated with HHT. β-catenin (green), DAPI (blue) staining and merged images indicate the nuclear translocation and expression of β-catenin. Scale bars, 100 μm. All data represent mean ± SEM.

Epithelial to mesenchymal transition is a prerequisite for cell migration and lies downstream of β-catenin^23^. Although previous studies have reported that Eph receptor is conducive to EMT progression in hepatoma cells^24^, the relationship between EphB4 and β-catenin has never been shown before. To investigate the role of β-catenin in HCC, we analyzed the mRNA level of β-catenin in HCC patients using The Cancer Genome Atlas (TCGA) database. RNASeq data from this database showed that *β-catenin* expression was significantly higher in carcinoma tissue compared with para-carcinoma tissue (Fig. 6a). Immunohistochemistry was used to detect β-catenin expression in 15 pairs of HCC and noncarcinoma tissues. The results showed that β-catenin expression was remarkably increased in carcinoma tissues compared with noncarcinoma tissues (Fig. 6b, c), which was consistent with the findings in TCGA database. To delineate the possible relationship between EphB4 and β-catenin, Spearman’s correlation analysis was conducted and the results revealed that *β-catenin* expression was positively correlated with *EphB4* levels in HCC tumor tissues (Fig. 6d).

We next analyzed the regulation of β-catenin in HepG2 cells exposed to HHT. Western blot analysis indicated that HHT could downregulate β-catenin expression and upregulate the phosphorylation of β-catenin level both in HepG2 cells and xenograft tumors (Figs. 6e, f and S2f, g). These results were also observed in immunohistochemical assay for xenograft tumors (Fig. 6g). And a remarkably reduced *β-catenin* mRNA level was also observed in HHT-treated HepG2 cells (Fig. S2h). Immunofluorescence staining was used to examine the distribution of β-catenin in HepG2 cells exposed to HHT. The results in Fig. 6h demonstrated that HHT restrained the level of β-catenin in the nucleus as well as in the cytoplasm. Figure 6e showed that phosphorylation of β-catenin was obviously increased at 20 and 40 nM of HHT, which has been reported to contribute to process of β-catenin degradation^25^. These data indicated that HHT suppressed nuclear translocation of β-catenin and promoted its phosphorylation.

### E-cadherin was overexpressed in HCC patients and HHT regulated EMT-related molecules

**Fig. 7:**
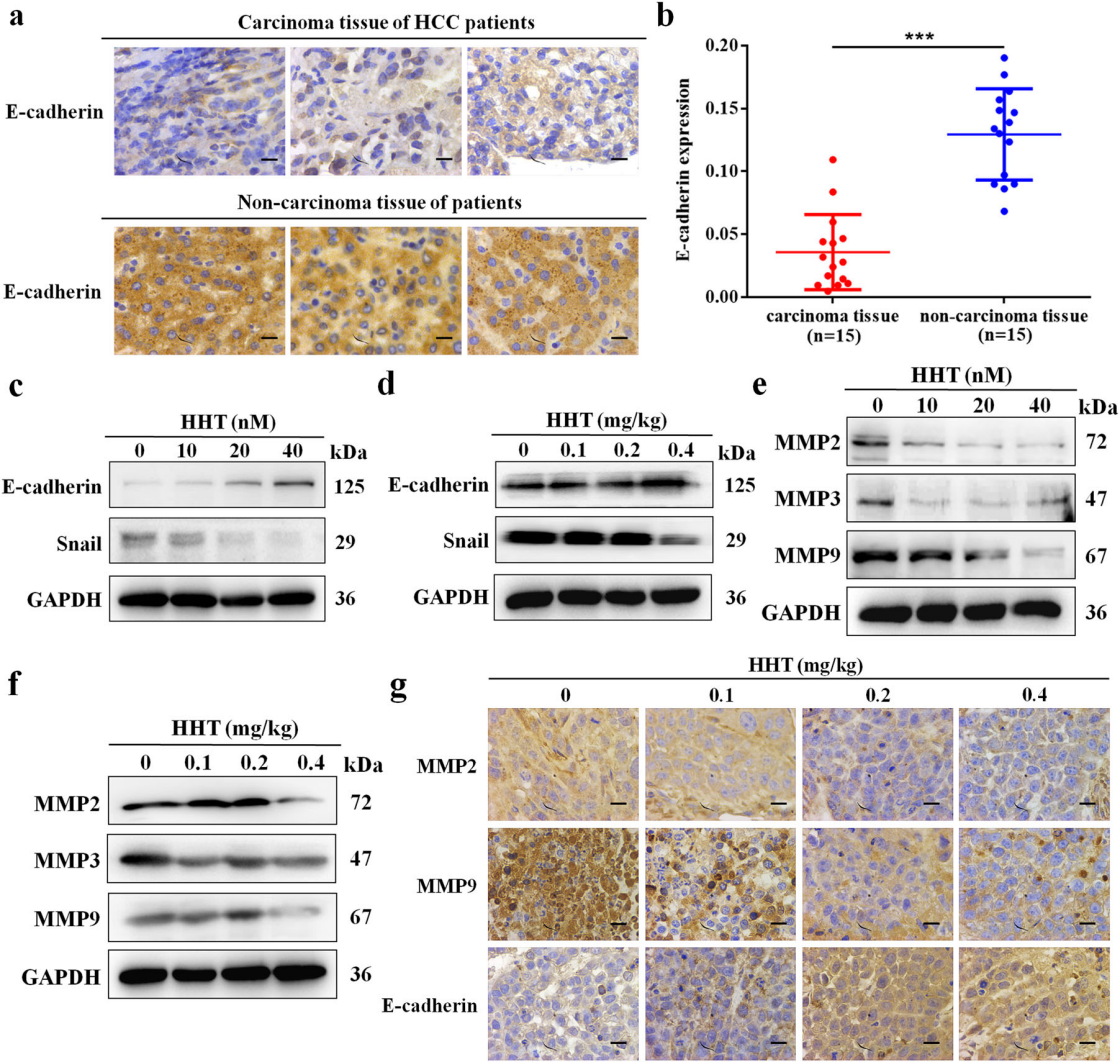
E-cadherin was overexpressed in HCC patients and HHT regulates EMT-related molecules. **a** Representative figures of immunohistochemical analysis of E-cadherin expression in carcinoma and noncarcinoma tissues derived from 15 HCC patients and 15 nonHCC patients, respectively. ×400 magnification. **b** Quantification of **a** (*n* = 15, ***p < 0.001). All data represent mean ± SEM. **c** Protein expression of E-cadherin and Snail in HepG2 cells treated with HHT for 48 h. **d** Protein expression of E-cadherin and Snail in HepG2 tumor EphB4 expression after HHT treatment. **e** Protein expression of MMP2, MMP3, and MMP9 in HepG2 cells treated with HHT for 48 h. **f** Protein expression of MMP2, MMP3, and MMP9 in HepG2 tumor tissues after HHT treatment. **g** Immunochemistry assay of E-cadherin, MMP2, and MMP9 in HepG2 tumor tissues. ×400 magnification.

Given the positive correlation of EphB4 and β-catenin in HCC patients, the E-cadherin expression in HCC patients was examined. As shown in Fig. 7a, b, lower E-cadherin protein was observed in carcinoma tissues, with higher expression in the noncarcinoma tissue group. Based on the result that E-cadherin was reduced in HCC patients and HHT could restrain the migration of HCC cells, we next analyzed the effect of HHT on EMT-related molecules by western blotting and immunohistochemistry assay. Promotion of E-cadherin and inhibition of Snail were observed in HHT-treated HepG2 cells and tumors (Figs. 7c, d, g and S3a, b). Furthermore, the results in Figs. 7e–g and S3c, d indicated that the essential members of MMPs family MMP2, MMP3, and MMP9 were suppressed by HHT both in HepG2 cells and in the tumor tissues of xenograft models. And the mRNA level of *MMP2* and *MMP9* were reduced in a dose depensent manner in HepG2 cells exposed to HHT (Fig. S3e).

### HHT repressed β-catenin and EMT-related molecules through EphB4 suppression

**Fig. 8:**
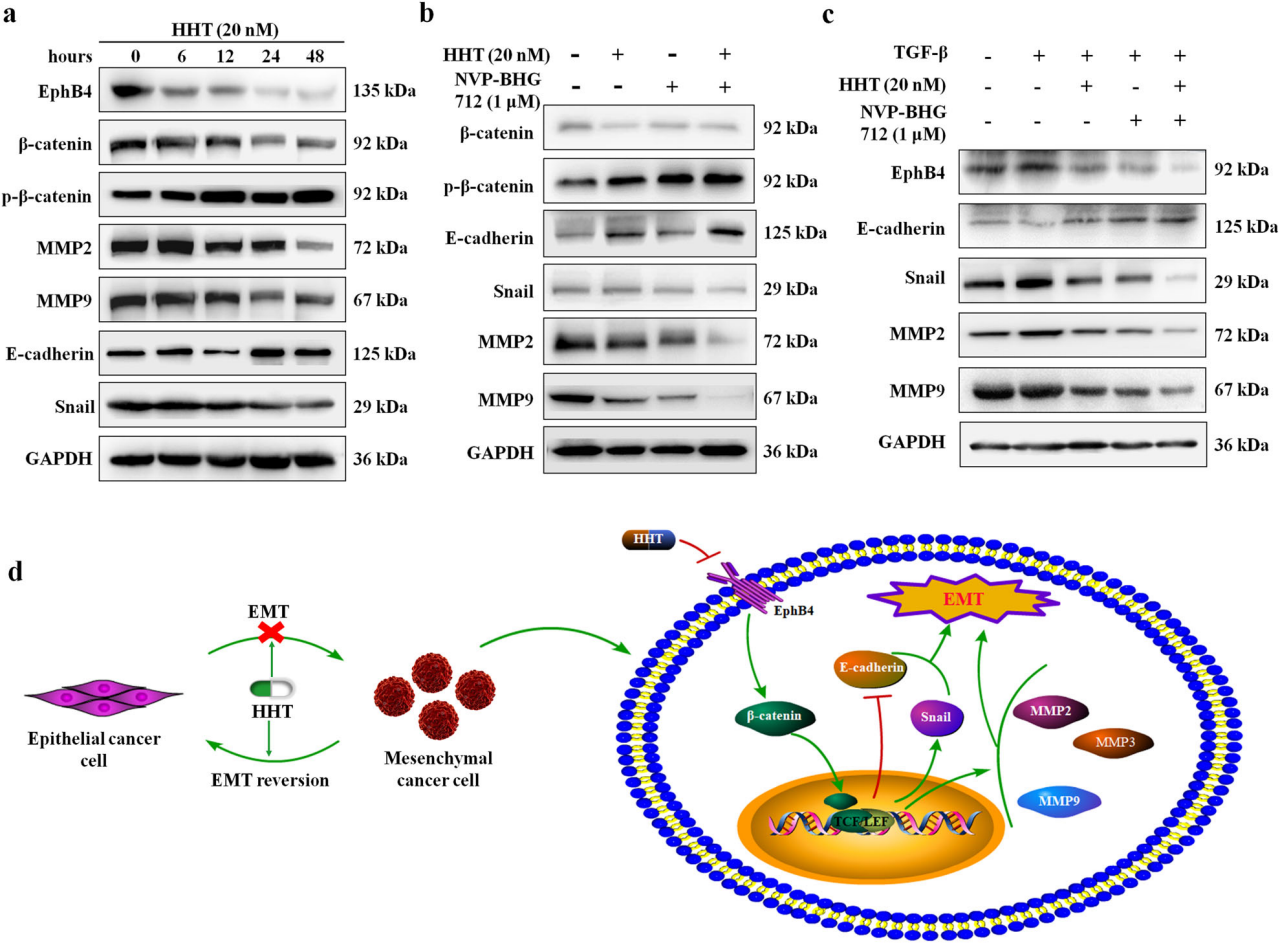
HHT repressed β-catenin and EMT-related molecules through EphB4 suppression. **a** Protein expression of β-catenin, p-β-catenin, E-cadherin, Snail, MMP2, and MMP9 in HepG2 cells treated with either: HHT (20 nM), NVP-BHG-712 (1 μM), or the combination of both. **b** Protein expression of EphB4, β-catenin, p-β-catenin, E-cadherin, Snail, MMP2, and MMP9 in HepG2 cells treated with HHT (20 nM) for the indicated duration. **c** Protein expression of EphB4, E-cadherin, Snail, MMP2, and MMP9 in HepG2 cells treated with either: vehicle, TGF-β, TGF-β + HHT, TGF-β + NVP-BHG712, or TGF-β + HHT + NVP-BHG712. **d** Schematic diagram of HHT inhibited the migration of HCC.

Next, the expression changes of EphB4, β-catenin, and EMT-related molecules after HHT administration for the different time points were evaluated by western blotting. The results in Figs. 8a and S4a–c demonstrated that the protein level of EphB4 was significantly decreased after HHT treatment within 6 h, and the expression of other molecules was unchanged at this time point. The expression and phosphorylation of β-catenin were remarkably changed within 12 h of HHT administration. Increased E-cadherin expression and decreased Snail, MMP2, and MMP9 expression were observed within 24 h of HHT treatment. These findings indicated that HHT might regulate the expression of β-catenin and EMT-related molecules by targeting EphB4 receptor. NVP-BHG712 was utilized to investigate the changes in these molecules after EphB4 suppression. The results in Figs. 8b and S4e demonstrated that both HHT and NVP-BHG712 could suppress β-catenin expression and promote its phosphorylation level. Furthermore, the upregulation of E-cadherin and downregulation of Snail, MMP2 and MMP9 were also seen in HHT or NVP-BHG712 monotherapy. These effects exerted by a single administration of HHT or NVP-BHG712 were significantly augmented by the combination of the two molecules.

EMT-related molecules in HepG2 cells following TGF-β stimulation was also investigated by western blot assay and the results were shown in Figs. 8c and S5a, b. The expression of E-cadherin was downregulated and the protein levels of Snail, MMP2, and MMP9 were upregulated by TGF-β, and these effects could be reversed by the addition of both HHT and NVP-BHG712. And concurrent addition of HHT and NVP-BHG712 further augmented the effect of monotherapy.

## Discussion

Continuous stimulation of proliferative signals and maladjustment of related monitoring mechanisms are important causes of tumor formation. The growth factor receptor can be activated by growth factors to generate intracellular cascade signals to regulate the proliferation of tumor cells. EphB4 is an important member of the receptor tyrosine kinase family which is overexpressed and conduces to tumor growth and migration in various cancers^6,13,15^. Our previous study has confirmed the overexpression of EphB4 in the tumor tissues of HCC patients, emphasizing EphB4 a potential target for HCC treatment^17^. However, there is no drugs targeting EphB4 on the market. In this study, we found the inhibitory effect of HHT on HCC cell proliferation and migration in an EphB4 dependent manner, and the underlying preliminarily mechanism was clarified.

HHT has been proved effective in the treatment of leukemia, but its potential in HCC inhibition was unknown. We revealed the antiproliferative ability of HHT on several HCC cell lines. In particular, HepG2 cells with the highest EphB4 protein expression were the most sensitive to HHT treatment, demonstrating that the inhibitory effect of HHT on HCC cells might be related to EphB4 expression. Xenograft models in nude mice confirmed the inhibitory effect of HHT on HepG2 cell growth in vivo. For in vitro experiments, EphB4 overexpression and EphrinB2 Fc stimulation increased the sensitivity of wild type HepG2 or Hep3B cells to HHT, while transient transfection with EphB4 siRNA decreased such effect in HepG2 cells. Similar results were drawn from in vivo experiments that HHT exhibited enhanced inhibitory effect in xenograft of EphB4^+^/7721 cells, compared to xenograft of wild type 7721 cells. The results above indicated that EphB4 might play an indispensable role in the suppression of HCC cell proliferation by HHT.

Invasion and migration are the main causes of tumor metastasis and the critical juncture of tumor staging in HCC^26,27^. Since EphB4 has been reported with promoting cell migration potential in both normal and malignant cells^7^, we investigate the role of EphB4 in cell migration suppression in HHT-treated HCC cells. Our results indicated that both EphB4 overexpression and exogenous stimulation with soluble EphrinB2 Fc exacerbated the antimigratory ability of HHT on SMMC-7721 cells both in wound healing and transwell migration assay. Furthermore, TGF-β, a multifunctional cytokine, was used to stimulate the migration ability of HepG2 cells^28^. The obtained results demonstrated that HHT restrained the migration of HepG2 cells stimulated by TGF-β, while EphB4 knockdown by siRNA impaired such inhibitory effect. Combined HHT and NVP-BHG712 treatment significantly augmented the antimigratory effect in TGF-β-stimulated HepG2 cells, as compared to either agent alone. Our further investigation confirmed that HHT was able to bind to EphB4 with hydrogen bonds and suppress its expression both in vitro and in vivo. These results indicated that HHT could inhibit cell migration by regulating EphB4 in HCC.

It has been reported that Eph receptor could mediate EMT progression and adhesion to conduce migratory and metastatic processes in hepatoma cells^24^. There is a wide acceptance that EMT is a prerequisite for cell migration and β-catenin can trigger EMT^23,29^, yet whether EphB4 could regulate β-catenin remains unknown. β-catenin was the key molecule of the Wnt/β-catenin pathway and the nuclear translocation of which could not only promote the expression of matrix metalloproteinases (MMPs) but also suppress E-cadherin expression^30,31^. In this study, both TCGA database and our own HCC patient samples analysis demonstrated that β-catenin was significantly overexpressed in HCC patients at protein and mRNA levels. We also analyzed the expression data of EphB4 and β-catenin in TCGA database and the results indicated that the mRNA level of the two molecules in HCC was significantly correlated, suggesting that β-catenin might play a critical role in HCC migration suppression by HHT. We examined the regulation of HHT on β-catenin and the results showed that HHT strikingly inhibited β-catenin expression at protein and mRNA level and promoted its phosphorylation in vitro and in vivo. Moreover, the result of immunofluorescence assay showed that the nuclear translocation of β-catenin was restrained by HHT.

As a key molecule of tumor migration, E-cadherin could be regulated by β-catenin, and the loss of E-cadherin is the critical marker of EMT^23,29^. We compared the protein expression of E-cadherin in the carcinoma tissues of HCC patients and the noncarcinoma tissues. The result indicated that E-cadherin level was prominently decreased in the carcinoma tissues compared to that in the noncarcinoma tissues. HHT treatment upregulated the protein level of E-cadherin both in HepG2 cells and xenograft tumors. Furthermore, Snail is a transcription factor and its expression is increased during the process of EMT. We found that Snail expression was significantly downregulated in HHT-treated cells and tumors. Most of the primary tumor cells are polar epithelial cells and connected to each other by intercellular adhesion molecules. As the tumor progresses, the intercellular adhesion molecules are degraded by MMPs^15^. Tumor migration-related molecules MMPs are the downstream signaling of the Wnt/β-catenin pathway and could be regulated by β-catenin. This study indicated that the expression of MMPs including MMP2, MMP3, and MMP9 was significantly suppressed by HHT in vitro and in vivo.

The obtained data showed that HHT could target EphB4 and suppress its expression. The expression of EphB4 and β-catenin in HCC was positively correlated according to TCGA data analysis. HHT treatment regulated the expression of β-catenin and its downstream signaling such as E-cadherin and MMPs. Next, we focused on the relationship between the effect of HHT on EphB4 and β-catenin and the downstream signaling. We investigated the protein levels in HepG2 cells exposed to HHT for different duration, and the results confirmed that β-catenin might be the downstream signaling of EphB4 receptor. EphB4 specific inhibitor NVP-BHG712 could suppress the protein level of β-catenin and promote its phosphorylation. The expression of E-cadherin, Snail, and MMPs was also significantly changed after EphB4 was suppressed by NVP-BHG712. And the regulating effect on EphB4, β-catenin and its downstream cascades was remarkably augmented in HepG2 cells after co-administration of HHT and NVP-BHG712. In addition, the increased expression of Snail and MMPs and decreased expression of E-cadherin confirmed that TGF-β induced EMT in HepG2 cells. Both HHT and NVP-BHG712 could reverse the regulating effect of TGF-β, and such effect was enhanced by combined HHT and NVP-BHG712 treatment. These findings indicated that HHT could reverse the EMT of HepG2 cells by restraining EphB4 expression, the suppression of which further inhibited the nucleus translocation of β-catenin and regulated the expression of EMT related molecules including E-cadherin, Snail, MMP2, and MMP9.

In conclusion, we discovered a positive correlation of EphB4 and β-catenin in HCC patients and that EphB4 was involved in HCC cell migration progression by regulating β-catenin-mediated EMT. HHT suppressed EphB4 expression and further led to β-catenin loss, resulting in the regulation of E-cadherin, Snail and MMPs to prevent EMT progression in HCC cells (Fig. 8d). Our research may provide new insight into the migration mechanism of EphB4 in HCC and HHT possesses great potential in the development of antiHCC drugs.

## Materials and methods

### Reagents

HHT (HPLC ≥ 98%) was obtained from Baoji Herbest Biotech Co., Ltd (Shaanxi, China). NVP-BHG712 (Purity ≥ 99%) was purchased from MedChemExpress Co., Ltd. Dulbecco’s modified Eagle’s medium (DMEM), RPMI 1640 medium and PBS were from HyClone (Logan, UT, USA). Fetal bovine serum (FBS) was purchased from ExCell Bio Co., Ltd (Shanghai, China). MTT powder, RNase and propidium iodide were from Sigma–Aldrich (St. Louis, MO, USA). Dimethyl sulfoxide (DMSO) was from Tianjin Kemiou Chemical Reagent Co., Ltd (Tianjin, China). Opti-MEM medium was purchased from Gibco (California, USA). Trypsin and Penicillin&Streptomycin solution were obtained from Beijing Solarbio Science & Technology Co., Ltd (Beijing, China). Lipofectamine 2000 reagent was purchased from Invitrogen (Carlsbad, CA, USA). RIPA Lysis Buffer and BCA protein assay reagent kit were purchased from Pioneer Biotechnology Co., Ltd. Protease and phosphatase inhibitors were obtained from Roche Tech. (Basel, Switzerland). Ultra Signal Enhanced Chemiluminescent (ECL) Reagent kit was purchased from 4A Biotech Co., Ltd (Beijing, China). EphB4, β-catenin, and p-β-catenin rabbit mAb were obtained from Cell Signaling Technology (Boston, MA, USA). E-cadherin, Snail, GAPDH rabbit mAbs, and goat antirabbit IgG were purchased from Protein technology Group (Chicago, Illinois, USA). MMP2, MMP3, and MMP9 rabbit mAb were from ABclonal (Boston, MA, USA). The EphB4 bacterial strain was from VectorBuilder.

### Patients

All the patients, who were eligible, underwent surgery at the First Affiliated Hospital of Xi’an Jiaotong University. Fifteen HCC tissues from HCC patients and fifteen hepatic tissues from nonHCC patients were obtained from consenting patients and used with permission of biomedical ethics committee of Xi’an Jiaotong University Health Science Center (Project No. 2019972).

### Cell culture

Human hepatocellular carcinoma HepG2, Hep3B, SMMC-7721 (7721), Bel-7402, and Bel-7404 cells were purchased from the Shanghai Institute of Cell Biology at the Chinese Academy of Sciences (Shanghai, China) without mycoplasma contamination. The HepG2 and Hep3B cells were cultured in DMEM with 10% FBS and 1% Penicillin and Streptomycin solution. SMMC-7721, Bel-7402, and Bel-7404 cells were cultured in RPMI-1640 with 10% FBS and 1% Penicillin and Streptomycin solution. All the cells were incubated in a humidified-atmosphere incubator of 5% CO_2_ at 37 °C.

### Cell viability assay

MTT method was used to analyze cell viability. The growing cells were seeded in 96-well plates overnight. Then the cells were treated with increased concentration of HHT for 48 h, followed by incubation with the mixture of serum free medium and MTT solution for 4–6 h. The mixture was replaced by DMSO. After 15 min shaking, the plates were determined using EPOCH (BioTek Instruments, Inc, USA) at a wavelength of 490 nm.

### Colony-forming assay

The growing cells were seeded in 12-well plates at a density of 300 cells per well. Following 24 h incubation, the cells were exposed to HTT for 48 h, followed by cultured in fresh complete medium for 2 weeks. After washed twice with PBS, the colonies were fixed by methanol and stained using 0.2% crystal violet. Images were obtained via an inverted fluorescence microscope.

### Migration assay

The HCC cells were cultured into the upper chamber at a density of 1 × 10^5^ cells per well, accompanied by 500 μL complete medium in the lower chamber. Following incubation for 24 h, for EphB4 plasmid transfection experiment, the 7721 cells were exposed to increased gradient of HTT (0, 0.25, 0.5, 1 μM) for 48 h. For EphB4 siRNA transfection experiment, the HepG2 cells were treated with TGF-β, TGF-β + HHT, TGF-β + NVP-BHG-712, or TGF-β + HHT + NVP-BHG-712, for 48 h. The medium was replaced with serum-free medium in the upper chamber and complete medium containing 20% FBS in the lower chamber. After 24 h incubation, a cotton swab was used to remove noninvading cells on the upper membranes of the upper chamber. The cells on the lower surface were fixed with 95% ethanol, stained with 0.1% crystal violet and counted. Images were obtained via an inverted fluorescence microscope.

### Wound healing assay

The growing cells were plated in 12-well plates with a total number of 1 × 10^6^ cells per well for 24 h. The cell monolayer was scratched a straight line using a sterile P200 pipet tip. After wash twice with PBS, for EphB4 plasmid transfection experiment, the cells were cultured in the fresh complete medium and treated with HTT for 24 h and 48 h, respectively. For EphB4 siRNA transfection experiment, the cells were treated with TGF-β and HHT, or TGF-β alone for 24 h and 48 h, respectively. The images were obtained at 0, 24, and 48 h using an inverted fluorescence microscope.

### Molecular docking

The ligand structure file was prepared by the Chemical draw software. The mol2 format structure file was converted to pdbqt format file using PyRx (Scripps research institute, USA). The EphB4 protein domain was confirmed by the interaction of NVP-BHG-712 and the EphB4 receptor protein (PDB ID: 6FNJ). A docking study was performed using Autodock Tools 1.5.6 to understand the molecular binding mode of HHT with EphB4 domain. The docking results were visualized using PyMOL 2.2.2 and Discovery Studio 4.5 software.

### EphB4 siRNA and plasmid transfection

A small interfering RNA of EphB4 was obtained from Shanghai GenePharma Co., Ltd. (Shanghai, China). EphB4 plasmid was extracted using an Endo-free Plasmid DNA Mini Kit I (OMEGA). Exponentially growing cells were seeded in 6-well plates overnight and treated with siRNA fragments or EphB4 plasmid using Lipofectamine 2000 reagent.

### Stimulation of cells with EphrinB2 Fc

EphrinB2 Fc (#C465) were obtained from Novoprotein. For Ephrin-B2 Fc stimulation, the serum-starved HCC cells were incubated with 4 μg/mL EphrinB2 Fc for 12 h

### Immunofluorescence staining

The growing HepG2 cells were plated into 96-well plates overnight and exposed to increased gradient of HHT (0, 10, 20, and 40 nM) for 48 h. Following fixed with 4% paraformaldehyde, the cells were blocked with 10% BSA at room temperature and incubated with β-catenin at 37 °C for 4 h. After rinsing with PBS, the cells were incubated with secondary antirabbit IgG antibody labeled with FITC for 1 h at room temperature, followed by staining with DAPI. Images were acquired using an inverted fluorescence microscope.

### Immunoblotting analysis

The cells or tumor tissues were lysed using RIPA buffer supplemented with protease and phosphatase inhibitor cocktail on ice for 30 min. The lysate was centrifuged at 12,000 × *g*/min for 10 min, and the supernatant was collected for immunoblotting analysis. A BCA Protein Quantification kit was used to confirm the concentration of the supernatant. Equivalent amounts of proteins were electrophoresed on SDS-polyacrylamide gels and transferred to polyvinylidene difluoride membranes. After blocking in 5% nonfat milk for 2 h, the membranes were incubated with the corresponding primary antibodies at 4 °C overnight, followed by rinsing with TBST. Then the membranes were incubated with HRP-conjugated secondary antibodies at 37 °C for 1 h, followed by rinsing with TBST. The result was detected using a Tanon 5200 automatic chemiluminescence image analysis system (Tanon, Shanghai, China) with an ECL kit.

### Immunohistochemistry assay

The tissues were fixed in 4% paraformaldehyde, embedded with paraffin, and sectioned into 5 μm-thick slices. Following deparaffinized in xylene, the slices were dehydrated in a 100, 95, 75% ethanol gradually, incubated with citrate buffer, and immersed in 3% hydrogen peroxide solution. Thereafter, the slices were incubated with the corresponding primary antibodies at 4 °C overnight and with the horseradish peroxidase (HRP)-conjugated secondary antibodies for 1 h at 37 °C. Finally, the sections were incubated with diaminobenzidine and re-dyed with hematoxylin for imaging.

### Quantitative reverse transcription PCR

The RNAfast200 RNA extraction kit was used to extract the total RNA according to the manufacture’s protocol. The revert aid first-strand cDNA synthesis kit was used to reverse-transcribe the total RNA samples as cDNA. Quantitative reverse transcription PCR was performed in 96-well reaction plates using the iCycleriQ RT-PCR detection system (Bio-Rad). The reaction solution contains cDNA, primers and SYBR Green Supermix. Thermal cycling conditions were as following: preincubation at 95 °C for 2 min, 40 PCR cycles at 95 °C for 20 s and at 60 °C for 1 min. All reactions run for three times. The relative amount of mRNA for each gene was normalized based on that of the gene β-actin. The primer sequences were as following: Human β-actin forward primer: 5′- CTCCATCCTGGCCTCGCTGT-3′; Human β-actin reverse primer: 5′- GCTGTCACCTTCACCGTTCC-3′; Human EphB4 forward primer: 5′- CAGGAACATCACAGCCAGAC-3′; Human EphB4 reverse primer: 5′- CAGGACCAGGACCACACC-3′; Human β-catenin forward primer: 5′- GGCAGCAACAGTCTTACCT-3′; Human β-catenin reverse primer: 5′- GTATCCACATCCTCTTCCTCAG-3′; Human MMP2 forward primer: 5′- GCCAACTACAACTTCTTCC-3′; Human MMP2 reverse primer: 5′- GCATCATCCACTGTCTCT-3′; Human MMP9 forward primer: 5′- CGGACCAAGGATACAGTT-3′; Human MMP9 reverse primer: 5′- AGTGAAGCGGTACATAGG-3′.

### Animals and xenograft models

Male nude mice (4–6 weeks of age) were used to conduct the animal studies. The mice were housed at Laboratory Animal Center of Xi’an Jiaotong University in a specific pathogen-free (SPF) atmosphere. All mice studies were performed according to regional authority guidelines and with permission of biomedical ethics committee of Xi’an Jiaotong University Health Science Center (Project No. 2019972). The number of animals in each group was calculated according the formula: *N* = 2[(*a* + *b*)^2^ *σ*^2^]/(*μ*_1_ − *μ*_2_)^2^. Each mouse was subcutaneously inoculated with 200 μL cell suspension (2 × 10^7^ cells/mL) at right flanks. The tumor volume was measured as *a* × *b*^2^/2, where a is the longer diameter and b is the shorter. For HepG2 cells, the tumor volume was measured once two days. When the tumor volume reached about 100 mm^3^, the mice were randomly divided into four groups (five mice each group) and administered intragastrically with HHT (0.1, 0.2, or 0.4 mg/kg) or 0.5% sodium carboxymethyl cellulose (CMC-Na) solution for 2 weeks. For 7721 or EphB4-overexpressing 7721 cells, the tumor volume was measured every day. The mice were randomly divided into two groups (four mice each group) and administered intragastrically with HHT (0.2 mg/kg) or 0.5% CMC-Na solution for 18 days. The body weights were monitored every day. Finally, the mice were euthanized, the tumors and spleens were weighed and imaged. Tumor tissues were harvested for immunohistochemistry and immunoblotting analysis.

### Statistical analysis

The values were evaluated as mean ± standard error of means. Statistical analysis was performed utilizing SPSS (version 16.0; IBM, Armonk, NY, USA). A student’s *t*-test was used to compare individual data with the control values. ANOVA was used to analyze statistical differences between groups under different conditions. Significance values were set at *p < 0.05.

## Supplementary information

Supplementary fig.1

Supplementary fig.2

Supplementary fig.3

Supplementary fig.4

Supplementary fig.5

Supplementary Figure Legends

## Data Availability

All data supporting the conclusions of this study are available within this article and the supplementary information.

## References

[1] Forner, A., Reig, M. & Bruix, J. Hepatocellular carcinoma. *Lancet* **391**, 1301–1314 (2018). 29307467 10.1016/S0140-6736(18)30010-2

[2] Qu, D., Cui, F., Lu, D., Yang, Y. & Xu, Y. DEP domain containing 1 predicts prognosis of hepatocellular carcinoma patients and regulates tumor proliferation and metastasis. *Cancer Sci.* **110**, 157–165 (2019). 30417471 10.1111/cas.13867 PMC6317931

[3] Xu, J. et al. Microwave responsive nanoplatform via P-selectin mediated drug delivery for treatment of hepatocellular carcinoma with distant metastasis. *Nano Lett.* **19**, 2914–2927 (2019). 30929452 10.1021/acs.nanolett.8b05202

[4] Tan, L. et al. Sublethal heat treatment of hepatocellular carcinoma promotes intrahepatic metastasis and stemness in a VEGFR1-dependent manner. *Cancer Lett.* **460**, 29–40 (2019). 31173855 10.1016/j.canlet.2019.05.041

[5] Jiang, Y. et al. Proteomics identifies new therapeutic targets of early-stage hepatocellular carcinoma. *Nature* **567**, 257–261 (2019). 30814741 10.1038/s41586-019-0987-8

[6] Ni, Q. et al. Expression levels of EPHB4, EFNB2 and caspase-8 are associated with clinicopathological features and progression of esophageal squamous cell cancer. *Oncol. Lett.* **19**, 917–929 (2020). 31885720 10.3892/ol.2019.11160 PMC6924202

[7] Chen, Y., Zhang, H. & Zhang, Y. Targeting receptor tyrosine kinase EphB4 in cancer therapy. *Semin. Cancer Biol.* **56**, 37–46 (2019). 28993206 10.1016/j.semcancer.2017.10.002

[8] Tan, Y. et al. Antitumor effects of circ-EPHB4 in hepatocellular carcinoma via inhibition of HIF-1alpha. *Mol. Carcinog.* **58**, 875–886 (2019). 30644610 10.1002/mc.22976

[9] Andolfo, I. et al. Kinome multigenic panel identified novel druggable EPHB4-V871I somatic variant in high-risk neuroblastoma. *J. Cell Mol. Med.* **24**, 6459–6471 (2020). 32336043 10.1111/jcmm.15297 PMC7294133

[10] Li, L., Nan, F., Guo, Q., Guan, D. & Zhou, C. Resistance to bevacizumab in ovarian cancer SKOV3 xenograft due to EphB4 overexpression. *J. Cancer Res. Ther.* **15**, 1282–1287 (2019). 31898661 10.4103/0973-1482.204896

[11] Ding, J. et al. Targeting the EphB4 receptor tyrosine kinase sensitizes HER2-positive breast cancer cells to Lapatinib. *Cancer Lett.* **475**, 53–64 (2020). 32006616 10.1016/j.canlet.2020.01.032

[12] Kadife, E. et al. Effects of EphB4 receptor expression on colorectal cancer cells, tumor growth, vascularization and composition. *Acta Oncol.* **57**, 1043–1056 (2018). 29368976 10.1080/0284186X.2018.1429650

[13] Salgia, R., Kulkarni, P. & Gill, P. S. EphB4: a promising target for upper aerodigestive malignancies. *Biochim. Biophys. Acta Rev. Cancer* **1869**, 128–137 (2018). 29369779 10.1016/j.bbcan.2018.01.003 PMC5955724

[14] Bhatia, S. et al. Inhibition of EphB4-Ephrin-B2 signaling reprograms the tumor immune microenvironment in head and neck cancers. *Cancer Res.* **79**, 2722–2735 (2019). 30894369 10.1158/0008-5472.CAN-18-3257 PMC6522285

[15] Xuqing, W. et al. EphB4 is overexpressed in papillary thyroid carcinoma and promotes the migration of papillary thyroid cancer cells. *Tumour Biol.* **33**, 1419–1427 (2012). 22528941 10.1007/s13277-012-0392-5

[16] Lennon, S. et al. Pancreatic tumor microenvironment modulation by EphB4-ephrinB2 inhibition and radiation combination. *Clin. Cancer Res.* **25**, 3352–3365 (2019). 30944125 10.1158/1078-0432.CCR-18-2811 PMC6548606

[17] Zhu, M. et al. Cantharidin treatment inhibits hepatocellular carcinoma development by regulating the JAK2/STAT3 and PI3K/Akt pathways in an EphB4-dependent manner. *Pharm. Res.* **158**, 104868 (2020). 10.1016/j.phrs.2020.104868 32407961

[18] Li, C. et al. Homoharringtonine exhibits potent anti-tumor effect and modulates DNA epigenome in acute myeloid leukemia by targeting SP1/TET1/5hmC. *Haematologica* **105**, 148–160 (2020). 30975912 10.3324/haematol.2018.208835 PMC6939512

[19] Chen, R. et al. Homoharringtonine reduced Mcl-1 expression and induced apoptosis in chronic lymphocytic leukemia. *Blood* **117**, 156–164 (2011). 20971952 10.1182/blood-2010-01-262808 PMC3037741

[20] Wolff, N. C. et al. High-throughput simultaneous screen and counterscreen identifies homoharringtonine as synthetic lethal with von Hippel-Lindau loss in renal cell carcinoma. *Oncotarget* **6**, 16951–16962 (2015). 26219258 10.18632/oncotarget.4773 PMC4627284

[21] Weng, T. Y. et al. Homoharringtonine induced immune alteration for an efficient anti-tumor response in mouse models of non-small cell lung adenocarcinoma expressing Kras mutation. *Sci. Rep.* **8**, 8216 (2018). 29844447 10.1038/s41598-018-26454-w PMC5974086

[22] Beranova, L. et al. The plant alkaloid and anti-leukemia drug homoharringtonine sensitizes resistant human colorectal carcinoma cells to TRAIL-induced apoptosis via multiple mechanisms. *Apoptosis* **18**, 739–750 (2013). 23456623 10.1007/s10495-013-0823-9

[23] Bure, I. V., Nemtsova, M. V. & Zaletaev, D. V. Roles of E-cadherin and noncoding RNAs in the epithelial-mesenchymal transition and progression in gastric cancer. *Int. J. Mol. Sci.* **20**, 2870 (2019). 10.3390/ijms20122870 PMC6627057 31212809

[24] Yan, Y. et al. MicroRNA-10a is involved in the metastatic process by regulating Eph tyrosine kinase receptor A4-mediated epithelial-mesenchymal transition and adhesion in hepatoma cells. *Hepatology* **57**, 667–677 (2013). 22996586 10.1002/hep.26071

[25] Li, H. et al. NCSTN promotes hepatocellular carcinoma cell growth and metastasis via beta-catenin activation in a Notch1/AKT dependent manner. *J. Exp. Clin. Cancer Res.* **39**, 128 (2020). 32631394 10.1186/s13046-020-01638-3 PMC7339515

[26] Liu, C. et al. Positive feedback loop of FAM83A/PI3K/AKT/c-Jun induces migration, invasion and metastasis in hepatocellular carcinoma. *Biomed. Pharmacother.* **123**, 109780 (2020). 31901550 10.1016/j.biopha.2019.109780

[27] Zuo, Q. et al. PGC1alpha suppresses metastasis of HCC by inhibiting Warburg effect via PPARgamma-dependent WNT/beta-catenin/PDK1 axis. Preprint at https://aasldpubs.onlinelibrary.wiley.com/doi/abs/10.1002/hep.31280 (2020).

[28] Zhang, S. et al. STIM1 and STIM2 differently regulate endogenous Ca(2+) entry and promote TGF-beta-induced EMT in breast cancer cells. *Biochem. Biophys. Res. Commun.* **488**, 74–80 (2017). 28479254 10.1016/j.bbrc.2017.05.009

[29] Zhou, P. et al. NMIIA promotes tumor growth and metastasis by activating the Wnt/beta-catenin signaling pathway and EMT in pancreatic cancer. *Oncogene* **38**, 5500–5515 (2019). 30967633 10.1038/s41388-019-0806-6

[30] Navarini, N. F. et al. Effect of epithelial growth factor on matrix metalloproteinase-2 and E-cadherin/beta-catenin expression in an in situ model of tumorigenesis. *Oncol. Lett.* **14**, 3136–3140 (2017). 28927057 10.3892/ol.2017.6513 PMC5588072

[31] Pantazi, E. et al. GLI2 is a regulator of beta-Catenin and Is associated with loss of E-Cadherin, cell invasiveness, and long-term epidermal regeneration. *J. Invest. Dermatol.* **137**, 1719–1730 (2017). 28300597 10.1016/j.jid.2016.11.046

