# Author Correction: Homoharringtonine suppresses tumor proliferation and migration by regulating EphB4-mediated β-catenin loss in hepatocellular carcinoma

**DOI:** 10.1038/s41419-024-07034-5

**Published:** 2024-09-02

**Authors:** Man Zhu, Zhengyan Gong, Qing Wu, Qi Su, Tianfeng Yang, Runze Yu, Rui Xu, Yanmin Zhang

**Affiliations:** School of Pharmacy, Health Science Center, Xi’an Jiaotong University, No. 76, Yanta Weststreet, #54, 710061 Xi’an, Shaanxi P.R. China

Correction to: *Cell Death and Disease* 10.1038/s41419-020-02902-2, published online 14 August 2020

In the original published version of this article, the authors unintentionally misplaced images of Fig. 4a and amended this by providing the correct image. This error does not affect any of the findings reported in the paper. The correct figure is shown below. The authors would like to apologize for any inconvenience this may have caused.

Amended Figure 4a
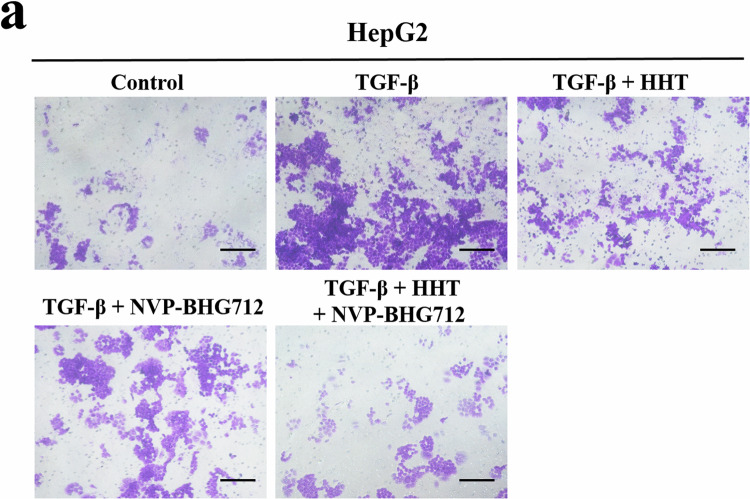


The original article has been corrected.

