# Supplementary figures and images for: Homoharringtonine suppresses tumor proliferation and migration by regulating EphB4-mediated β-catenin loss in hepatocellular carcinoma
Source: Cell Death Dis. 2020 Aug 14;11(8):632. doi: 10.1038/s41419-020-02902-2 (PMC7429962; doi:10.1038/s41419-020-02902-2)

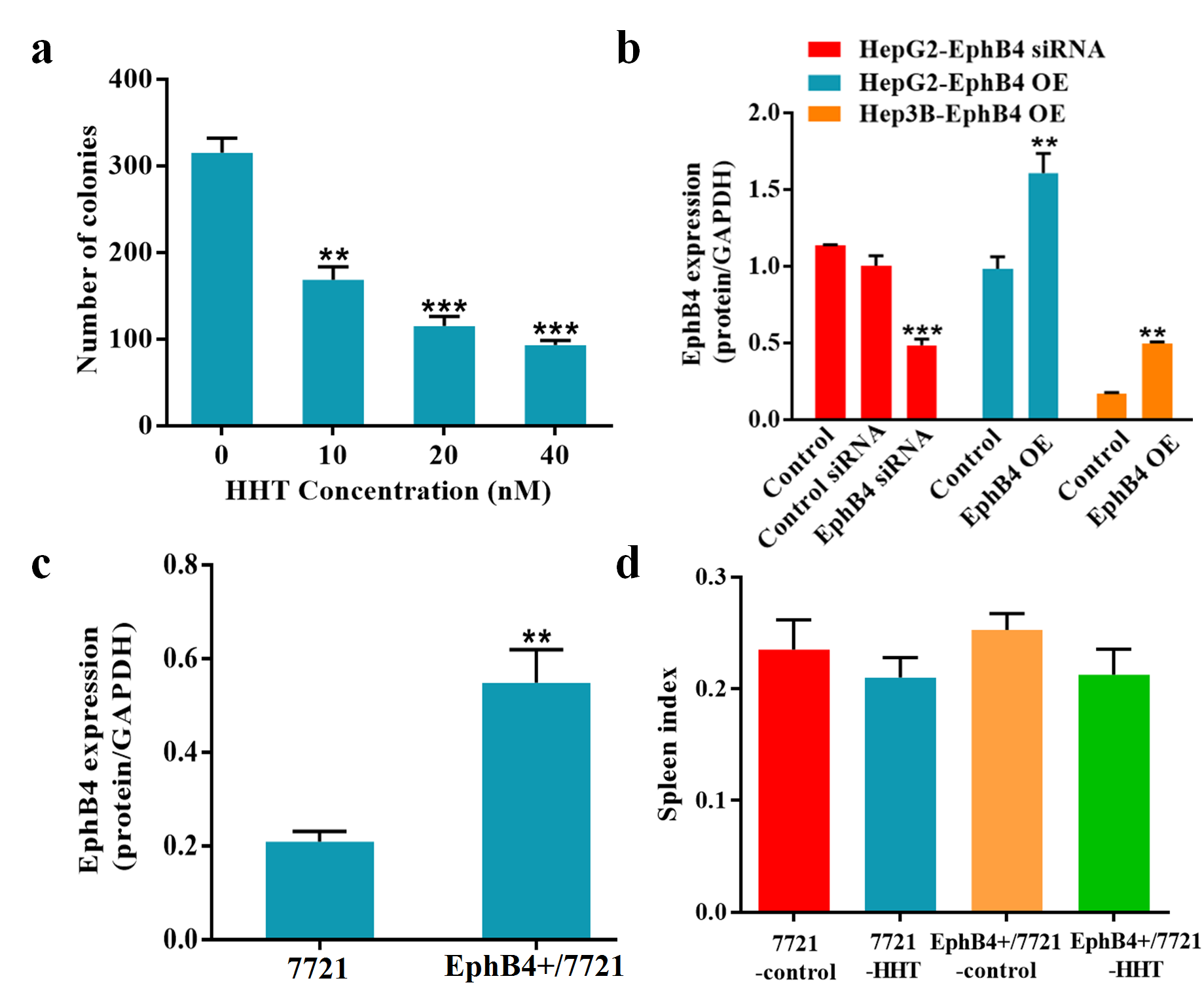

Supplement: Supplementary file 1 — Supplementary fig.1 [file 41419_2020_2902_MOESM1_ESM.tif]

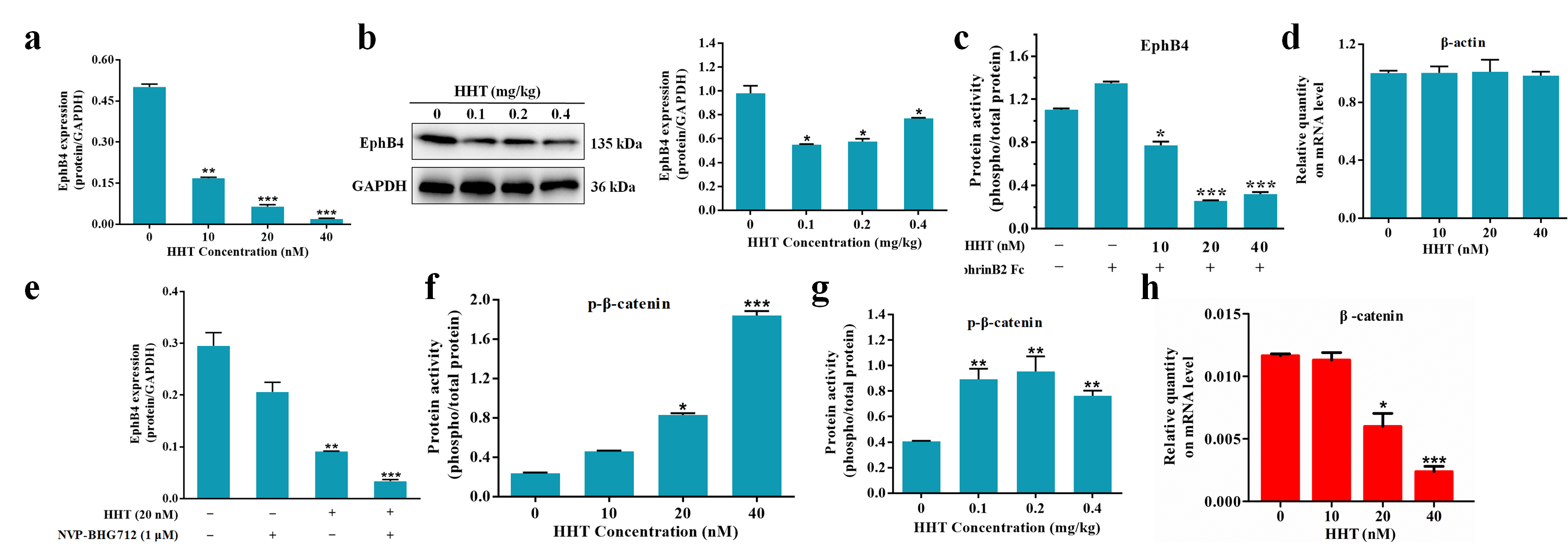

Supplement: Supplementary file 2 — Supplementary fig.2 [file 41419_2020_2902_MOESM2_ESM.tif]

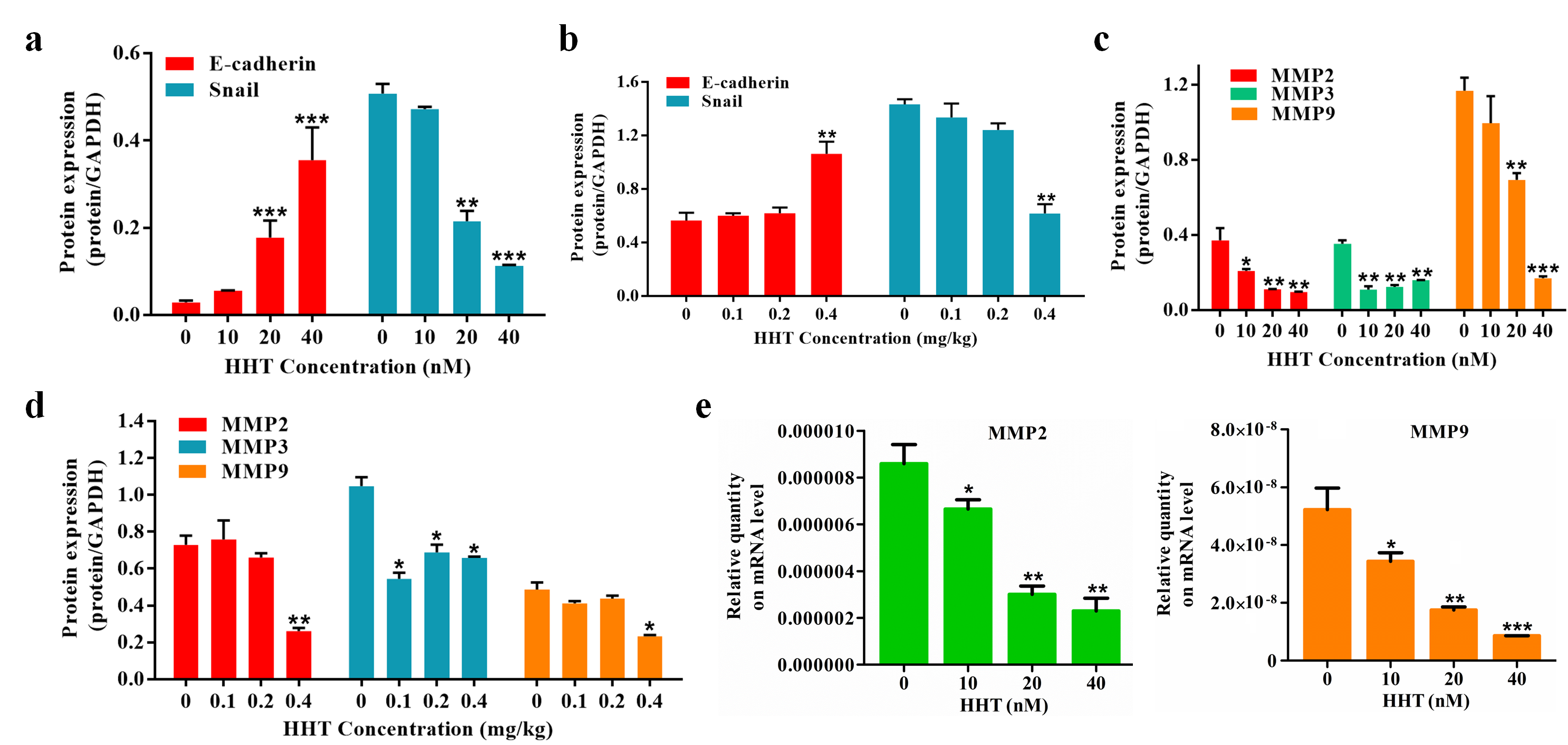

Supplement: Supplementary file 3 — Supplementary fig.3 [file 41419_2020_2902_MOESM3_ESM.tif]

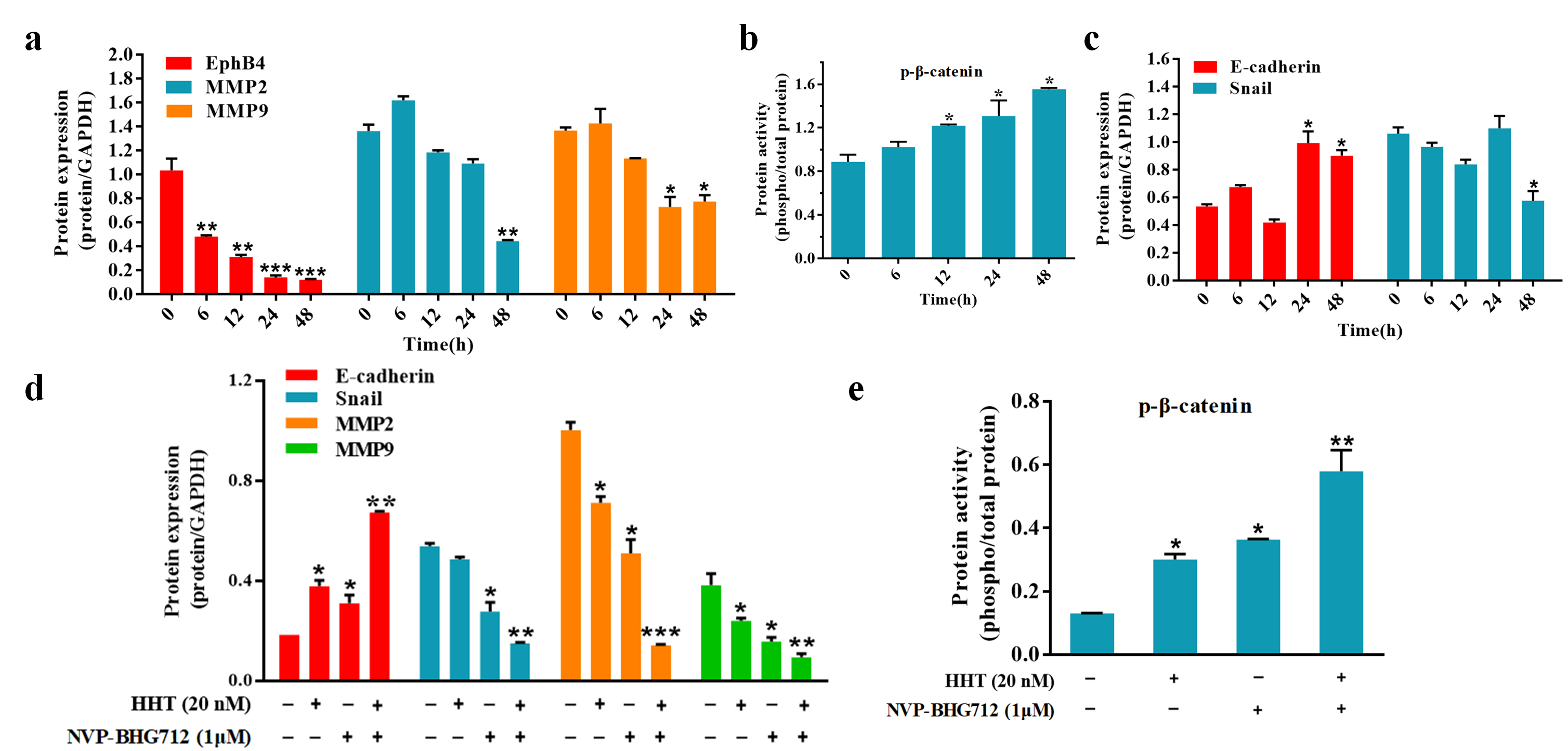

Supplement: Supplementary file 4 — Supplementary fig.4 [file 41419_2020_2902_MOESM4_ESM.tif]

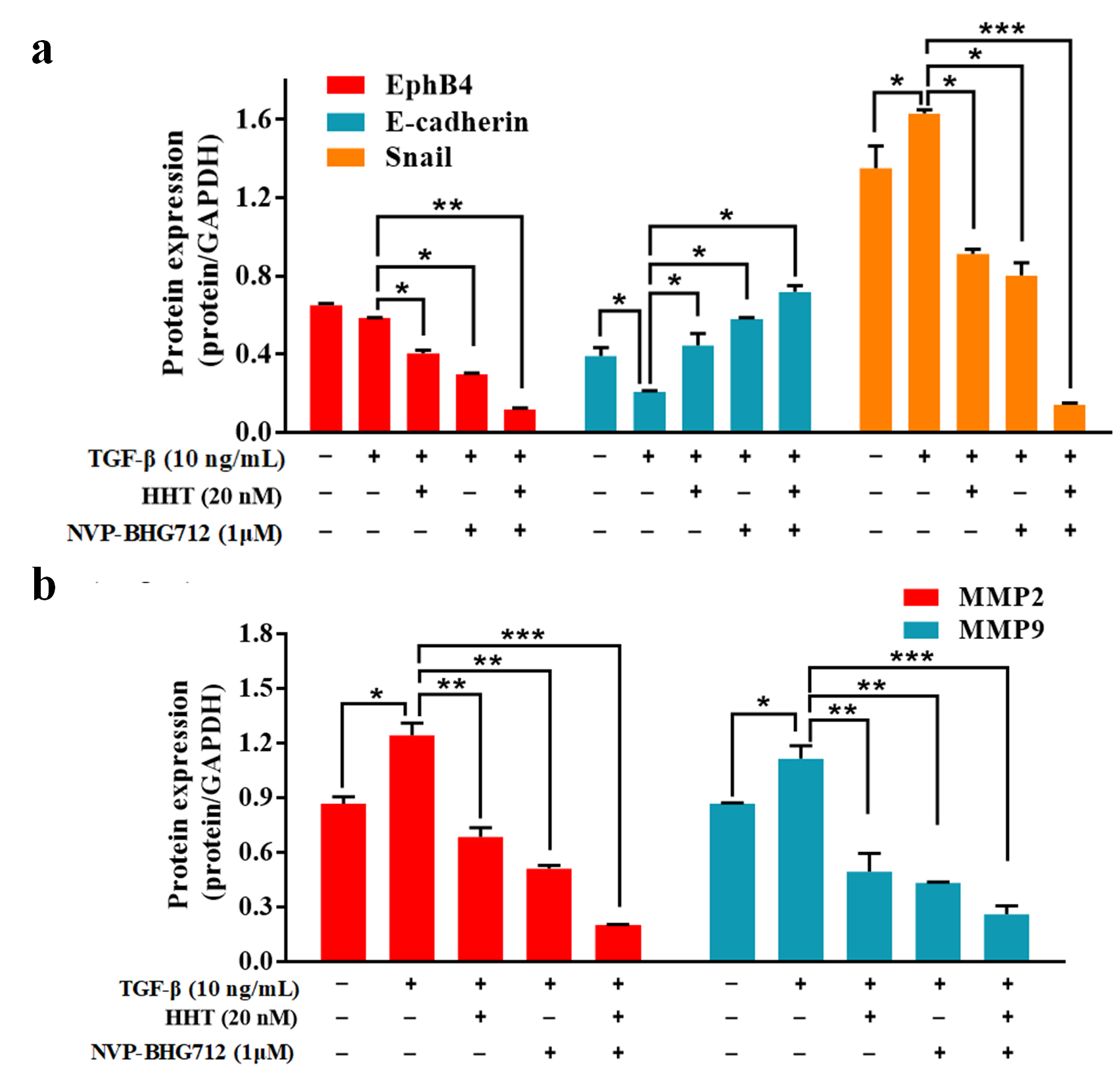

Supplement: Supplementary file 5 — Supplementary fig.5 [file 41419_2020_2902_MOESM5_ESM.tif]
