# Supplementary material for: Homoharringtonine suppresses tumor proliferation and migration by regulating EphB4-mediated β-catenin loss in hepatocellular carcinoma
Source: Cell Death Dis. 2020 Aug 14;11(8):632. doi: 10.1038/s41419-020-02902-2 (PMC7429962; doi:10.1038/s41419-020-02902-2)
Supplement: Supplementary file 6 — Supplementary Figure Legends [file 41419_2020_2902_MOESM6_ESM.docx]

**Supplementary Figures Legends**

Fig. S1 **a** Quantification of Fig. 1**e** (n = 5). **b** Quantification of Fig. 2**a, c** (n = 3). **c** Quantification of Fig. 2**e** (n = 3). **d** Spleen index of control and HHT-treated group mice (n = 4). All data represent mean ± SEM. **p <*0.05, ***p* <0.01, and ****p* <0.001; compared to vehicle controls.

Fig. S2 **a** Quantification of Fig. 3**a** (n = 3). **b** Protein expression of EphB4 in HepG2 tumor tissues after HHT treatment (n = 3). **c** Quantification of Fig. 5**b** (n = 3). **d** RT-PCR analysis of HepG2 cells β-actin expression after HHT treatment (n = 5). **e** Quantification of Fig. 5**e** (n = 3). **f** Quantification of Fig. 6**e** (n = 3). **g** Quantification of Fig. 6**f** (n = 3). **h** RT-PCR analysis of HepG2 cells β-catenin expression after HHT treatment (n = 5). All data represent mean ± SEM. **p <*0.05, ***p* <0.01, and ****p* <0.001; compared to vehicle controls.

Fig. S3 **a** Quantification of Fig. 7**c** (n = 3). **b** Quantification of Fig. 7**d** (n = 3). **c** Quantification of Fig. 7**e** (n = 3). **d** Quantification of 7**f** (n = 3). **e** RT-PCR analysis of HepG2 cells MMP2 and MMP9 expression after HHT treatment (n = 5). All data represent mean ± SEM. **p <*0.05, ***p* <0.01, and ****p* <0.001; compared to vehicle controls.

Fig. S4 **a-c** Quantification of Fig. 8**a** (n = 3). **d, e** Quantification of Fig. 8**b** (n = 3). All data represent mean ± SEM. **p <*0.05, ***p* <0.01, and ****p* <0.001; compared to vehicle controls.

Fig. S5 **a, b** Quantification of Fig. 8**c** (n = 3). All data represent mean ± SEM. **p <*0.05, ***p* <0.01, and ****p* <0.001; compared to vehicle controls.
